# Chronic Treatment With Psilocybin Decreases Changes in Body Weight in a Rodent Model of Obesity

**DOI:** 10.3389/fpsyt.2022.891512

**Published:** 2022-05-18

**Authors:** Joyce Huang, Michelle Pham, William J. Panenka, William G. Honer, Alasdair M. Barr

**Affiliations:** ^1^Department of Anesthesiology, Pharmacology and Therapeutics, Faculty of Medicine, University of British Columbia, Vancouver, BC, Canada; ^2^Department of Psychiatry, Faculty of Medicine, University of British Columbia, Vancouver, BC, Canada; ^3^British Columbia Mental Health & Substance Use Services Research Institute, Vancouver, BC, Canada

**Keywords:** adiposity, animal model, obesity, psilocybin, psychedelic, weight gain

## Abstract

**Background:**

There are currently relatively few effective pharmacological treatments for obesity, and existing ones may be associated with limiting side-effects. In the search for novel anti-obesity agents, drugs that modify central serotonergic systems have historically proven to be effective in promoting weight loss. Psilocin, which is rapidly metabolized from psilocybin, is an agonist at multiple serotonin receptors. In the present study we assessed the effects of psilocybin and a positive control (metformin) on changes in body weight in a rat model of obesity.

**Methods:**

Five groups of adult male rats were pre-conditioned with a cafeteria diet until obese (>600 g) and then treated with either psilocybin (0.1, 1, or 5 mg/kg, i.p.), metformin (300 mg/kg, p.o.) or vehicle control. Treatments were for 27 consecutive weekdays, and body weights and high calorie food intake were recorded daily. Fasting glucose levels were recorded after 11 days of treatment. At the end of treatment rats completed a glucose tolerance test, and multiple fat pads were dissected out to assess adiposity.

**Results:**

The medium dose psilocybin group had to be terminated from the study prematurely. Both the low and high dose psilocybin groups caused a significant decrease in changes in body weight compared to controls. The metformin group produced a greater decrease in change in body weight than either psilocybin groups or controls. Both high dose psilocybin and metformin decreased consumption of the high calorie diet, and exhibited decreased central adiposity.

**Conclusion:**

Psilocybin demonstrated modest but significant effects on weight gain. Further study is recommended.

## Introduction

Pharmacological treatments for obesity exert their therapeutic effects on a diverse range of physiological targets. Historically, one of the most successful substrates for anti-obesity drug development has been the central serotonergic system (1–3). The monoamine neurotransmitter serotonin plays a key role in multiple processes related to feeding, and mediates central effects on appetite and satiety *via* both hypothalamic and extrahypothalamic brain structures (4). Thus, drugs which elevate synaptic levels of serotonin by either direct release (amphetamines, fenfluramine) or by reuptake inhibition (sibutramine, tesofensine) have been proven to exhibit efficacy in promoting weight loss (5, 6). However, this class of drugs has been associated with a number of serious side-effects, which has resulted in their withdrawal from most markets (7). To reduce the risk of off-target effects caused by non-specific elevations of serotonin levels, likely mediated through the 14 known serotonin receptors (8), there has been a focus on a more targeted approach using receptor-specific ligands (9). The comprehensive preclinical literature suggests that at least the 5-HT1B, 5-HT2C, and 5-HT6 receptors play an important role in satiety, and hold promise for therapeutic development of weight-loss drugs (4). For example, the 5-HT2C agonist lorcaserin proved to be an effective treatment for obesity before its recent withdrawal in the US due to concerns about an increased risk of cancer (10).

The serotonergic system remains a promising substrate for developing new anti-obesity medications, despite the list of previously-withdrawn compounds–especially if products can be identified that have minimal affinity for receptors leading to serotonergic side-effects, such as the 5-HT2B receptor which has been associated with valvulopathies (11). One potential compound that mediates its downstream effects *via* serotonin receptors, and which has been used extensively by humans, is the prodrug psilocybin. Found naturally in numerous species of fungi, psilocybin has been used for centuries by many cultures for religious and recreational purposes (12). Interestingly, recent analysis of adults who have used serotonergic psychedelics (including psilocybin) showed significantly lower odds of being overweight/obese or having cardiometabolic diseases, with higher odds of self-reported overall health (13), although the mechanisms underlying these observations remain to be understood. Importantly, psilocybin has seen a surge of interest in the field of psychiatry due to evidence of a rapid and sustained antidepressant effect (14). Evidence indicates a bidirectional association between depression and obesity (15), and several of the pharmacological treatments used for the former show benefits in the latter, in preclinical and clinical studies (7). It remains to be seen whether serotonergic psychedelics may have weight modulating properties which warrant further study as a potential therapeutic modality. In particular, psilocybin is well-suited as a candidate to study as it is considered to be amongst the safest of all the psychedelic drugs with regards to somatotoxic and psychological side-effects (16, 17).

The purpose of the present study was therefore to determine whether chronic treatment with psilocybin would exert effects on weight gain in an animal model of obesity. Rodent paradigms of obesity have strong predictive validity with regards to drug development, in which most compounds that affect weight gain in rodents also affect weight in humans, and vice versa (18, 19). We used an established model of obesity in which rodents are voluntarily fed a high calorie “cafeteria” diet and rapidly gain weight (20). For dosing psilocybin, we included two common doses of the drug (1 and 5 mg/kg) as well as a “microdose” (0.1 mg/kg) (21). The rationale for the latter dose is the emerging interest in ingesting lower doses of psychedelics that have sub-perceptible effects (typically about 1/10th of their recreational dose) (22–25).

## Materials and Methods

### Animals

Ninety male, adult Sprague-Dawley rats (275–300 g) were obtained from Charles River (Montreal, QC). The animals were allowed to habituate in the UBC animal colony before being exposed to the high calorie diet. Rats were randomly housed in pairs in large polycarbonate cages on ventilated racks in a reverse-light cycle, temperature-controlled (22 ± 1°C) room and maintained on a 12-h light-dark cycle (lights on at 07:00 h). All rats, except one control group, were provided regular rat-chow and were given *ad libitum* access to both the rat-chow and the cafeteria diet to reach a desired weight (≈600 g) prior to experimental procedures. Cafeteria diets were prepared freshly and leftover diet food was removed and weighed daily (weekdays). Animals were familiarized to handlers through being weighed every Monday, Wednesday, and Friday for at least 30 days prior to drug treatment. The animals were all experimentally naïve, and stratified according to their body weight to obtain body weight-matched treatment groups. Animals were treated daily Monday–Friday, to be consistent with a recent study which used psilocybin to reduce alcohol seeking behavior in rats (21), and had a total of 27 drug treatment days; access to the colony was restricted on weekends due to safety measures related to the global COVID-19 pandemic. This latter point meant that regular rat chow was required to be changed on a daily basis by animal care staff, and so could not be measured by those running the present experiments. Animal were also monitored for signs of general health on a daily basis, as required in the protocol by the REB. This included any changes in neurological function (e.g., seizure) or general behavior, respiration, hydration/elimination, and any signs of stress and/or pain. All experimental procedures were conducted in accordance with the National Institutes of Health Guide for the Care and Use of Laboratory Animals, and approved by The University of British Columbia’s Animal Care and Use Committee.

### Pharmaceutical Agents and Solutions

Three doses of psilocybin dissolved in sterile saline (0.9%) were used: low (0.1 mg/kg), medium (1.0 mg/kg), and high (5.0 mg/kg). Psilocybin (99.7% purity; Psygen Labs Inc., Calgary, AB, Canada) was prepared under sterile conditions by the Katz Group at the Rexall Centre for Pharmacy & Health Research at the University of Alberta. Metformin (300 mg/kg; Toronto Research Chemicals) was slightly warmed during mixing to aid in dissolution. All solutions were prepared fresh daily, and vortexed immediately prior to loading the syringe to ensure administration of a homogenous solution. Each animal received either a 1 ml/kg oral gavage of metformin, or an intraperitoneal (IP) injection of saline or the dose of psilocybin (0.1, 1, and 5 mg/kg) dissolved in a volume of 1 ml/kg of sterile saline solution. IP injection sites were rotated through the week to prevent infection and irritation (26). Psilocybin was administered by IP injection rather than *via* oral gavage (like metformin) due to the dearth of published studies on oral administration of the drug, resulting in challenges determining an optimal dose to exert behavioral effects.

### Cafeteria Diet

The highly palatable high-fat and high-sugar diet was composed of, by weight, 10.9% crushed cheddar crackers (Goldfish: 25% fat, 65% carbohydrate, 10% protein), 53% chocolate hazelnut spread (Nutella: 31% fat, 58% carbohydrate, 6% protein) and 36% smooth peanut butter (Kraft: 47% fat, 20% carbohydrate, 27% protein). An additional 4 mini-marshmallows (100% carbohydrate) and 6g crushed chips (Ruffles regular: 38% fat, 54% carbohydrate, 7% protein) were added Monday–Thursday, and 6 mini-marshmallows and 8 g crushed chips were added on Friday. The food components were mashed into a coarse paste as per previously reported for high calorie diets (27–29), and thus the different food components were consumed in proportion to their contribution of the above makeup. The exception to this was the mini-marshmallows, which did not dissolve into the paste and so could be counted the following day if left unconsumed. Nutritional and calorie content were obtained from the commercial websites for the different food products.

### Blood Collection and Intraperitoneal Glucose Tolerance Test

Glucose measurements were collected from each rat prior to the start of drug treatment, after 10 or 11 days of drug treatment, and at the end of the study. All animals were fasted overnight prior to blood collection. Blood was collected from the lateral saphenous vein, as previously (30). Glucose measurements were obtained using a handheld glucometer (One Touch Ultra) (31). At the end of the study, an intraperitoneal glucose tolerance test (IGTT) was performed, as previously described (32). All animals were fasted overnight, then given a glucose challenge of 1 g/ml/kg glucose administered by IP injection. Blood glucose levels were then measured at 60 and 120 min following glucose injection.

### Dissection

At the end of the study, the rats were euthanized by inhalant anesthetic (isoflurane), followed by decapitation to collect the brains for future studies. Fat deposits (subcutaneous [defined area], left retroperitoneal and left perirenal) were excised and weighed, as previously reported (33).

### Statistical Analysis

Longitudinal data, including daily weight gain and food consumption, were analyzed for group and time differences using repeated-measures analysis of variance (ANOVA) (SPSS Version 24.0. Armonk, NY: IBM Corp). Non-longitudinal data were compared using independent-samples t-test or one-way ANOVA, as previously (34). Associations between variables were determined using the Pearson correlation coefficient. Comparison of categorical variables between groups where cells had less than five counts used Fisher’s exact test. Significance for all statistical tests was set at *p* < 0.05. *Post-hoc* tests were analyzed using the Fisher’s LSD test.

## Results

Treatments were well-handled by the animals, who habituated quickly to the daily procedures. Rats were fed the cafeteria diet for a total of 51 days to get animals to the desired weight. At baseline, immediately prior to drug treatment, all groups previously exposed to the cafeteria diet exhibited significantly greater weight than the control group given only standard rat-chow [*F*_(5,87)_ = 3.11, *p* < 0.05], with a mean weight of 609.7 g for cafeteria-fed and 548.6 g for standard chow-only animals.

At the approximate midpoint of the study, one treatment group (the medium dose of psilocybin) was accidentally treated with an incorrect drug treatment, and so treatment was discontinued for this group; their data are included at the mid-point analysis, for informative reasons only.

Change in body weight over the 27 days of treatment was analyzed in relative terms (measured by change in body weight relative to baseline body weight, expressed as a percentage). Similarly, food consumption was measured in relative terms (food consumed in proportion to body weight, expressed as a percentage).

**FIGURE 1:**
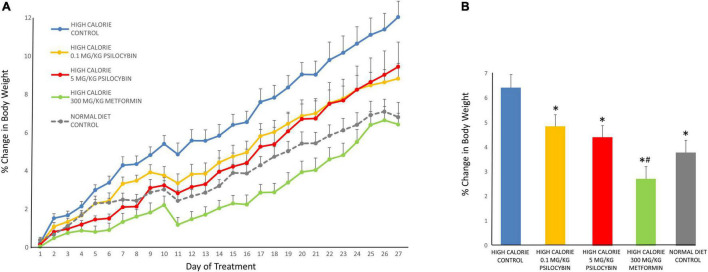
Effects of chronic psilocybin or metformin on change in relative body weight. **(A)** Data demonstrate change in relative body weight on a daily basis after 27 weekday treatments in rats fed either a high calorie diet or regular rat chow. Values represent percentage change in body weight relative to body weight at baseline (i.e., the final day before drug treatment). Adult male rats (*n* = 14–16 per group) were treated chronically with low (0.1 mg/kg i.p.) or high (5 mg/kg i.p.) doses of psilocybin, metformin (300 mg/kg p.o.) or vehicle (0.9% saline solution). Values are group means ± SEM. **(B)** Data represent mean change in relative body weight averaged across the 27 treatments. Values represent percentage change in body weight relative to body weight at baseline (i.e., the final day before drug treatment). Values are group means ± SEM. *Statistically different from high calorie diet control group, *p* < 0.05; **^#^**Statistically different versus both psilocybin groups, *p* < 0.05.

When relative weight gain was analyzed, the repeated measures ANOVA indicated a significant main effect of drug treatment [*F*_(4,68)_ = 6.92, *p* < 0.001], a main effect of time [*F*_(26,1768)_ = 261.359, *p* < 0.001] and a drug by time interaction [*F*_(104,1768)_ = 3.391, *p* < 0.001]. The *post-hoc* analysis revealed that when relative weight gain was compared across all 27 treatment days, the greatest relative weight gain was exhibited by the control group, fed the high calorie diet (Figure 1). The low dose psilocybin group demonstrated less relative weight gain; this was a significant effect compared to the control group (*p* < 0.05). The high dose psilocybin group exhibited less relative weight gain too, and this effect was also statistically significant (*p* < 0.01). The metformin group displayed the least relative weight gain compared to all of the other groups fed with the high calorie diet (*p* < 0.05). Exploratory *post-hoc* analyses on day-by-day changes in body weight indicated a rapid effect of psilocybin treatment, whereby relative weight gain was less in the medium and high dose psilocybin groups—as well as the metformin group—by only the second day of treatment. The low dose of psilocybin group exhibited significantly lower weight gain compared to the high calorie control group by the sixth day of treatment.

**FIGURE 2:**
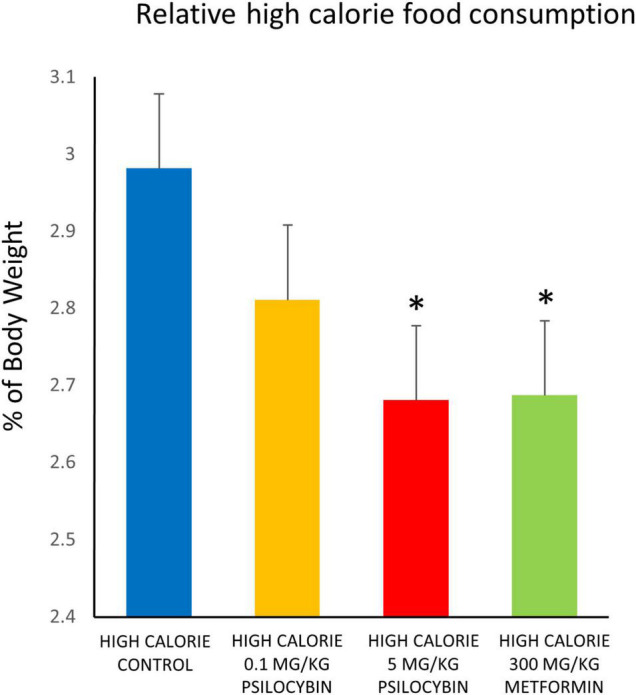
Effects of chronic psilocybin or metformin on mean daily food consumption, as percentage of body weight. Data represent mean consumption of high calorie cafeteria diet, expressed as percentage of body weight, averaged across the 27 days of treatment. Rats were fed the high calorie cafeteria diet during weekdays only. Amount of high calorie diet food (excluding regular rat chow) consumed after each day of treatment was measured (g) and calculated as% of individual body weight (g) for that particular day. Adult male rats (*n* = 14–16 per group) were treated chronically with low (0.1 mg/kg i.p.) or high (5 mg/kg i.p.) doses of psilocybin, metformin (300 mg/kg p.o.), or vehicle (0.9% saline solution), and fed high calorie diet. Does not account for consumption of regular rat chow. Values are group means ± SEM. *Statistically different from high calorie diet control group, *p* < 0.05.

With regards to food consumption, when relative amount of food consumed was analyzed the repeated measures ANOVA indicated a non-significant main effect of drug treatment [*F*_(3,55)_ = 1.990, *p* = 0.126], a main effect of time [*F*_(24,1320)_ = 4.782, *p* < 0.001] and a non-significant drug by time interaction. Follow up exploratory analyses based on *a priori* predictions indicated that both the metformin and high dose psilocybin groups ate relatively less of the high calorie food than the control animals over the entire treatment period (*p* < 0.05) (Figure 2). Because the analysis of weight change indicated that drug treatment had relatively rapid effects, we conducted a follow-up analysis of relative food consumption in shorter time bins (days 1–6, 7–13, 14–19, and 20–27). Interestingly, relative amount of food consumed differed significantly between groups from days 1–6 [*F*_(4,73)_ = 3.12, *p* < 0.01] and was a non-significant trend for days 7–13 [*F*_(4,73)_ = 2.32, *p* = 0.06], but was not significant in the latter half of the study. *Post-hoc* tests indicated that the high calorie diet control group consumed more food (3.22 ± 0.14% of body weight [mean ± SEM]) than all other groups given the cafeteria diet (low dose psilocybin: 2.79 ± 0.13%; high dose psilocybin: 2.81 ± 0.13%; metformin 2.64 ± 0.14% of body weight) from days 1–6, and significantly more than the high dose psilocybin and metformin groups from days 7–13 (controls: 3.19 ± 0.14%; low dose psilocybin: 2.91 ± 0.13%; high dose psilocybin: 2.63 ± 0.13%; metformin 2.72 ± 0.14% of body weight).

Consumption was also analyzed based on caloric content, rather than weight, for the total amount of food consumed over the duration of the high calorie diet following drug treatment. The control group treated with saline solution consumed the most calories (4430.9 ± 221.5 kcal), followed by the low dose psilocybin (4145.9 ± 211.5 kcal), then the high dose psilocybin (3955.5 ± 233.2 kcal) and then least of all the metformin group (3705.7 ± 130.9 kcal). Similar to the relative amount of food consumed, the ANOVA did not find an overall group effect of drug treatment [*F*_(3,55)_ = 2.04, *p* = 0.120], but based on *a priori* predictions from the amount of food consumed, it was indicated that the metformin group consumed significantly fewer calories than the vehicle control group (*p* < 0.05). Because the high calorie diet was a relatively homogeneous paste, it was not possible to determine whether the drug treatments could have caused a selective reduction in specific macronutrient (fat, carbohydrate or protein) intake, which might have been possible had the food products remained distinct. Of interest, however, only the metformin treated group had leftover mini-marshmallows the following day (115 total marshmallows over the course of drug treatment, vs. none for all of the other groups; *p* < 0.001).

Analysis of fasting glucose levels at the approximate mid-point of treatment did not indicate a significant main effect of group [*F*_(5,87)_ = 1.736, *p* = 0.136] indicating the groups did not differ in their fasting glucose levels (Table 1). However, when all of the high calorie diet animals were analyzed as one group and compared to the normal diet group, there was a non-significant trend [*t*_(86)_ = 1.720, *p* = 0.089], indicating a modest increase in fasting glucose levels for high calorie diet animals.

**TABLE 1:** Fasting glucose levels at treatment day 11 and during intraperitoneal glucose tolerance test (IGTT) at treatment day 27.

Group	Fasting glucose day 11	IGTT 1 h Day 27	IGTT 2 h day 27
High calorie diet Control	6.09 ± 0.20	8.04 ± 0.41	6.10 ± 0.20
High calorie diet Low dose psilocybin	6.34 ± 0.17	8.11 ± 0.46	6.65 ± 0.23
High calorie diet Medium dose psilocybin	5.80 ± 0.15	ND	ND
High calorie diet High dose psilocybin	5.96 ± 0.22	8.13 ± 0.41	6.56 ± 0.26
High calorie diet Metformin	5.94 ± 0.14	7.81 ± 0.38	6.38 ± 0.32
Normal diet Control	5.69 ± 0.15	6.24 ± 0.22*	5.84 ± 0.18**^#^**

Fasting glucose levels reported as (mmol/L); adult male rats (n = 14–16 per group) were treated chronically with low (0.1 mg/kg i.p.), medium (1 mg/kg i.p.), or high (5 mg/kg i.p.) doses of psilocybin, metformin (300 mg/kg p.o.) or vehicle (0.9% saline solution), and fed either high calorie diet or standard rat chow. Overnight fasting glucose levels were recorded on Day 11 at the approximate midpoint of the study, and then on the final day of treatment Day 27 at 1 and 2 h after a glucose challenge in the IGTT. Values are group means ± SEM. *Statistically different from all other groups at 1 h timepoint of IGTT, p < 0.05; **^#^**Statistically different from low and high dose psilocybin groups at 2 h timepoint of IGTT, p < 0.05. ND = not determined.

The analysis of glucose sensitivity with the intraperitoneal glucose tolerance test at the end of treatment at the one and 2 h time points by repeated measures ANOVA revealed a group effect of drug treatment [*F*_(4,68)_ = 3.326, *p* < 0.05], a main effect of time [*F*_(1,68)_ = 118.734, *p* < 0.001] and a drug by time interaction [*F*_(4,68)_ = 4.094, *p* = 0.005] (Table 1). *Post-hoc* analysis indicated that the significance was largely driven by the normal diet group, which exhibited significantly lower glucose levels than all of the other groups, particularly at the 1 h time point. None of the groups in the high calorie diet group differed from each other. The normal diet group exhibited the typical pattern of glucose clearance observed in non-obese Sprague-Dawley rats [e.g., as observed in our laboratory (35)], with mildly elevated glucose levels at 1 h after glucose challenge, and a return to baseline levels by 2 h.

When the weights of different fad pads at the end of treatment were analyzed, normalized to body weight (to determine relative adiposity), two of the three fat regions exhibited significant group differences (Table 2). While there was no group difference for perirenal fat [*F*_(4,72)_ = 0.995, ns], the subcutaneous [*F*_(4,72)_ = 3.089, *p* < 0.05] and retroperitoneal fat [*F*_(4,72)_ = 10.282, *p* < 0.001] differed by group. *Post-hoc* analysis indicated that for the subcutaneous fat, the normal diet differed from all of the high calorie fed groups with the exception of the metformin group. For the retroperitoneal fat, the normal diet, high dose psilocybin and metformin groups had lower relative adiposity than the high calorie control group.

**TABLE 2:** Relative adiposity for different fat pads following 27 days of treatment.

Group	Subcutaneous fat	Retroperitoneal fat	Perirenal fat
High calorie diet Control	4.89 ± 0.39	2.37 ± 0.23	0.53 ± 0.09
High calorie diet Low dose psilocybin	4.41 ± 0.25	2.17 ± 0.12	0.53 ± 0.09
High calorie diet High dose psilocybin	4.80 ± 0.39	1.93 ± 0.14**^#^**	0.41 ± 0.03
High calorie diet Metformin	4.22 ± 0.20	1.90 ± 0.09**^#^**	0.49 ± 0.09
Normal diet Control	3.47 ± 0.31*	1.12 ± 0.11**^#^**	0.34 ± 0.08

Relative adiposity, expressed as percentage of total body weight (%) for subcutaneous fat (a defined area), left retroperitoneal and perirenal fat pads; adult male rats (n = 14–16 per group) were treated chronically with low (0.1 mg/kg i.p.) or high (5 mg/kg i.p.) doses of psilocybin, metformin (300 mg/kg p.o.) or vehicle (0.9% saline solution), and fed either high calorie diet or standard rat chow. Fat pads were dissected out at the end of the experiment using standard procedures in our laboratory. Values are group means ± SEM. *Statistically different from all groups other than metformin, P < 0.05; **^#^**Statistically different high calorie diet control group, p < 0.05.

Exploratory associations between weight, feeding and metabolic indices indicated that there was a significant correlation between both subcutaneous (*r* = 0.528, *p* < 0.001) and retroperitoneal fat weights (*r* = 0.544, *p* < 0.001) and glucose intolerance at the 1 h timepoint, but not for the perirenal fat (*r* = 0.091, NS). Total relative weight gain correlated significantly with subcutaneous and retroperitoneal adiposity, but not perirenal fat. Unsurprisingly, total relative weight gain correlated strongly (*r* = 0.666, *p* < 0.001) with total amount of high calorie diet consumed.

## Discussion

In the present study, we evaluated the effects of chronic treatment with three different doses of psilocybin (including a “microdose”), as well as a single dose of the oral antidiabetic drug metformin, on change in body weight in a rat model of obesity. Animals received 27 continuous weekday treatments with the drug or its vehicle. An accidental error of drug administration with the medium dose group (1 mg/kg) resulted in their early termination from the study. Psilocybin was well-tolerated by the other two treatment groups, with no obvious evidence of harmful effects, based on standard daily laboratory monitoring procedures. The principal finding of the study was that both doses of psilocybin resulted in significantly decreased relative weight gain compared to high calorie diet-fed rats treated with the control vehicle; metformin also caused decreased relative weight gain, with a greater magnitude than either of the psilocybin doses. Both the high dose psilocybin and metformin groups ate less of the high calorie diet—these effects were most pronounced in the first 2 weeks of treatment. There were no effects of any drug treatment on fasting glucose levels or glucose sensitivity in the IGTT. However, both the high dose psilocybin and metformin groups had lower relative central adiposity than the high calorie control group, and this correlated with glucose levels in the IGTT.

These overall findings suggest that psilocybin may have modest weight-loss properties, which could feasibly extend to humans. While none of the groups in this study exhibited an actual reduction in absolute weight over the period of treatment compared to their baseline weight, this may reflect a limitation of the current model species. Laboratory rats will typically continue to gain weight throughout their lifetime, until at least 24–32 months of age (36), when they can weigh over 1 kg (37). In models of diet-induced obesity, high calorie fed control rats will usually continue to gain weight throughout the study, and so decreased weight gain compared to controls will often be interpreted as a “weight-loss” effect, even if there is an absolute increase in mass over time [e.g., Hansen et al. (38)]. In the context of obese humans who are maintaining a stable weight, the slowed weight gain in psilocybin-treated rats could therefore be anticipated to correspond to a weight-loss in individuals treated with the drug, rather than just a delay of any further weight gain. In support of this, both the high calorie fed group that received metformin and the control group fed normal rat chow (with no access to the high calorie diet) also continued to gain weight, with the latter group showing equivalent weight gain to the two psilocybin groups. Notably, metformin has well-established weight loss properties in obese humans (39), and so the manner in which the drug was only able to slow weight gain in the present study, rather than cause absolute weight-loss, again suggests that potential weight-loss effects in humans should be inferred from *relative* changes in the weight of drug-treated animals compared to control rats.

The significantly greater effect of metformin than the two doses of psilocybin on change in body weight may indicate that the weight-altering effects of the latter drug are less effective, although uncertainties exist around equivalent dosing between the two drugs and their relative weight-altering effects in humans. The dose of metformin used in the present study would be considered at the higher end of appropriate dosing for an animal study (40, 41), while the maximal physiologically relevant dose for psilocybin remains less well-established (42). Our dosing schedule was based on that used in a recent behavioral rat study of the effects of psilocybin on alcohol-seeking behavior, in which their dosing was determined in consultation with experts in the field, who opined that the minimum dose needed to see behavioral effects in rats is 1 mg/kg (21). The study by Meinhardt et al. (21) used repeated doses of psilocybin as high as 10 mg/kg. Thus, our “medium” dose may actually reflect a minimally sufficient dose to alter behavior, while our “high” dose may correspond more closely to a mid-range value. But it is also worth considering how the present doses compare to those used in human clinical trials, based on allometric comparisons between species. The typical dose of psilocybin used in landmark clinical trials for conditions such as depression is 25 mg (43, 44), corresponding to a dose of approximately 0.3 mg/kg in humans. Using validated allometric guidelines (45), the current psilocybin doses of 0.1, 1, and 5 mg/kg equate to 0.016, 0.16, and 0.81 mg/kg Human Equivalent Doses. Additionally, the administration of psilocybin *via* IP injection might be expected to result in greater bioavailability than by oral consumption. Thus, it is also possible that the 5 mg/dose of psilocybin may represent a higher dose than is typically used in humans. Future studies will therefore be needed with higher doses of psilocybin to determine whether larger doses are able to affect weight gain equivalently to a high dose of metformin before final comparisons can be made between drug efficacy for weight-loss, possibly combined with pharmacokinetic indices to determine the validity of such doses to human trials.

Of interest, we did not observe any effect of drug treatment—including metformin–on glucose levels either during a fast or following a glucose challenge in the high calorie diet animals. This may be partly because the magnitude of the effects of the diet on glucose dysregulation were modest: differences between the high calorie and regular diet groups were only significant when all animals from the high calorie diet were included as one large group. This small effect size therefore restricted the statistical power of drug treatment to produce a significant reduction in glucose values. It is important, thus, to emphasize that the current animal model is one of obesity, rather than Type 2 diabetes (T2DM). Rodent models of diet-induced obesity typically do not result in the development of diabetes (18) although they can exhibit other types of metabolic dysregulation. The present model was chosen because it utilized a cafeteria diet model that included widely available supplies, is easy to dispense and measure, is economical, and does not change from day-to-day (27). The model was also effective previously in detecting the weight loss properties of both sibutramine and liraglutide (27) with chronic treatment—two drugs that promote weight loss in humans. Like the study by Hansen et al. (27), our animals did not develop frank diabetes. However, in their study the cafeteria diet fed animals did demonstrate more pronounced glucose dysregulation in the GTT. As their rats were approximately 130 g heavier at the start of the study, it is possible that the current starting weight of animals in our study was not yet sufficiently large enough to maximize metabolic dysregulation. Nevertheless, it will be important to test the effects of psilocybin using more complex paradigms of obesity and metabolic syndrome/diabetes, using models such as the Zucker rat (46) to assess the entirety of metabolic changes that might be improved.

The mechanism(s) by which psilocybin slowed weight gain in the high calorie diet rats will require further study, as they were beyond the scope of the present series of experiments. It is possible that an important part of the effect is due to reduced intake of the high calorie diet—this was statistically significant for both the high dose psilocybin and metformin groups, but not the low dose psilocybin group, indicating that additional factors may be involved. Due to restrictions around weekend access to the colony, animal care staff were in charge of providing regular rat chow on a daily basis (which was co-provided with the high calorie diet, to ensure that animals’ nutritional needs were met) and thus consumption of regular chow could not be measured by research staff, nor could the high calorie cafeteria diet be provided to animals on these days. It is possible that the low dose psilocybin group selectively reduced their consumption of the less palatable regular chow rather than the high calorie diet; while this option seems counterintuitive, it will need to be dispelled by future experiments that measure the consumption of all food sources. The pharmacology of psilocybin is certainly consistent with effects on feeding behavior. Psilocybin is rapidly dephosphorylated in the body by alkaline phosphatase to the active metabolite psilocin (47). Both *in vitro* and *in vivo* studies have confirmed that psilocin is an agonist at 5-HT1A, 5-HT2A, and 5-HT2C receptors with moderate-to-high affinity (48–51). Of these, the 5-HT2C receptor has been demonstrated repeatedly to play a key role in satiety (52), and treatment with 5-HT2C agonists results in weight-loss while 5-HT2C antagonists cause weight gain (4, 53–55). 5-HT2C agonists may also reduce feeding through other mechanisms, including suppressing conditioned responding and impulsivity (56), which will require more complex animal feeding models to evaluate for psilocybin. In addition to effects on feeding, there is also a substantial body of evidence which indicates that 5-HT2C agonists can produce effects on blood glucose levels and insulin sensitivity independent of changes in weight and feeding (1). Interestingly, these drugs (such as meta-chlorophenylpiperazine and lorcaserin) were able to improve glucose homeostasis at doses well below those needed to reduce food intake (57, 58). This may be relevant to the present study, where we observed that treatment with the low “microdose” (0.1 mg/kg) of psilocybin—at a dose that is considered only 1/10th that needed to alter behavior in rats (21)—was able to significantly decrease weight gain in the absence of any change in consumption of the high calorie diet. It is also possible that psilocybin may have altered weight by affecting metabolism and resting energy expenditure (59). Ongoing studies are presently addressing these possibilities.

While the present findings represent a novel study of the potential effects of psilocybin on obesity, there are a number of important limitations. Firstly, we are not able to provide any insight into the molecular mechanisms that are involved in decreased weight gain. The current study was designed as an exploratory examination of the effects of psilocybin on obesity, with further study contingent on an observable effect. This phenomenon has now been produced twice in our laboratory (a pilot study showed similar effects) and so studies to determine the neural/metabolic substrates involved are proceeding. Secondly, we only studied the effects of psilocybin in male rats. Rat models of obesity typically use male rather than female animals to avoid the complicating effects of the estrous cycle (18, 60), but female rodents exhibit meaningful differences in energy homeostasis and adiposity (61, 62), and so it will be critical to determine whether similar benefits are observed with female rats too. Thirdly, as noted above, we may have potentially under-dosed with psilocybin at the higher end of our dosing regimen. If effects are dose-dependent, it will be important to assess where the maximal effects of psilocybin peak on weight gain at physiologically relevant doses. Use of psilocybin is complicated by regulatory requirements, access to high purity product, and (typically) cost, but chronic studies with higher doses of the drug may be necessary to make further advances in this area. However, as noted above, allometric comparison of the current doses in rats indicates that the 5 mg/kg dose may already be exceeding the Human Equivalent Dose typically used in clinical trials. Therefore, pharmacokinetic measures (such as plasma levels of psilocybin/psilocin) may be helpful in cross-species comparison to determine if higher doses are justified, as well as use of alternate behavioral assays in rodents of serotonergic activity [such as the head twitch response/wet dog shakes (63)] in parallel. Finally, while not necessarily a limitation, the present study used repeated, chronic treatment with psilocybin, whereas most human trials typically use a much small number of doses, often in combination with psychotherapy (43, 44). Repeated exposure to psychedelic drugs can result in behavioral tolerance (64) and downregulation of the 5-HT2A receptor (65). This may have even occurred in the present study, as decreased food intake diminished with time more rapidly with the psilocybin than metformin groups. Thus, intermittent treatment with psilocybin may represent a future direction of study to maximize potential weight loss by preventing behavioral tolerance.

In summary, the present study provides preliminary findings from a rodent model of obesity that psilocybin has potential weight reducing properties, which are relatively fast onset. Obesity represents a huge unmet medical need in many countries (66), with approximately 40% of the US population being affected (67). A recent study noted that drugs used to treat T2DM were prescribed 15 times more frequently than those used specifically for obesity (68). This reflects in part the small number of drugs indicated for obesity (six currently approved by the US Food and Drug Administration: phentermine/topiramate, naltrexone/bupropion, liraglutide, semaglutide, orlistat, and setmelanotide) (69) and the issue of side-effects as well as cost. The potential of psilocybin as a weight-loss tool, whereby it could be consumed orally—possibly at a non-psychoactive dose, and therefore on a more frequent basis—may have value, even if weight loss effects were only modest; it is important to reinforce that none of the doses of psilocybin used in our study exerted effects as strong as those observed with metformin. Issues remain about the drug’s safety, as we are not aware of any large-scale studies of chronic treatment with the drug, but concerns about the cardiac valvular problems associated with earlier serotonergic drugs may be less warranted, given psilocin’s lesser affinity for the 5-HT2B receptor (50). Psychological side-effects of psilocybin treatment may also be lessened if “microdoses” could be used to obtain weight-loss benefits. These possibilities suggest that further study on this topic should proceed.

## Data Availability Statement

The datasets presented in this article are not readily available because the present data serve as the basis for multiple patent applications. Data will be made available upon approval of patents. Requests to access the datasets should be directed to AB, al.barr@ubc.ca.

## Ethics Statement

The animal study was reviewed and approved by UBC Animal Care Committee.

## Author Contributions

JH and MP ran the study and collected the data. AB designed the study, conducted the data analysis, and wrote the first draft of the manuscript. WP and WH assisted in interpretation of results. All authors contributed to the final version of the manuscript.

## Conflict of Interest

WH has received consulting fees or sat on paid advisory boards for the Canadian Agency for Drugs and Technology in Health, AlphaSights, Guiepoint, *in silico*, Translational Life Sciences, Otsuka, Lundbeck, and Newron. WP was the founder and CEO of Translational Life Sciences, an early stage science company focused on psychedelic-based therapeutics. AB served as a consultant to Translational Life Sciences. The remaining authors declare that the research was conducted in the absence of any commercial or financial relationships that could be construed as a potential conflict of interest.

## Publisher’s Note

All claims expressed in this article are solely those of the authors and do not necessarily represent those of their affiliated organizations, or those of the publisher, the editors and the reviewers. Any product that may be evaluated in this article, or claim that may be made by its manufacturer, is not guaranteed or endorsed by the publisher.
